# Draft Genome Sequence of *Tepidimonas taiwanensis* Strain VT154-175

**DOI:** 10.1128/genomeA.00942-16

**Published:** 2016-09-08

**Authors:** Federica Valeriani, Tommaso Biagini, Saverio Giampaoli, Silvia Crognale, Daniele Santoni, Vincenzo Romano Spica

**Affiliations:** a Public Health Unit, University of Rome “Foro Italico,” Rome, Italy; b Mendel Institute, Rome, Italy; c DIBAF, University of Tuscia, Viterbo, Italy; d Institute for System Analysis and Computer Science “Antonio Ruberti,” National Research Council, Rome, Italy

## Abstract

The slightly thermophilic bacterium *Tepidimonas taiwanensis* strain VT154-175 has been isolated from a hot spring in the area of Viterbo, Italy. The whole draft genome of 2.9 Mb obtained by paired-end next-generation sequencing and divided into 60 scaffolds is presented.

## GENOME ANNOUNCEMENT

The microbial characterization of hot springs can be particularly important for understanding the first steps of life on primordial Earth, as well as for the discovery of novel taxa useful for advances in many biotechnological fields (1, 2). Bacteria belonging to the genus *Tepidimonas* are slightly thermophilic bacteria found in hot springs (3–6). In particular the species *Tepidimonas taiwanensis* has been isolated in hot springs in southern Taiwan (7), Romania, and Central India (8, 9). In addition, *Tepidimonas* spp. were found in Italy in industrial water circuits with temperatures at 40°C and above (10).

In the present study, the draft genome sequences of *T. taiwanensis* strain VT154-175 are described. The selected strain was isolated from a Bullicame hot spring (GPS: 42°25′15′′N, 12°3′52′′E; water temperature: 54°C) close to the city of Viterbo, Italy, and previously described (11, 12). Microbiological isolation has been performed on agar plate with medium D modified (13, 14). The cultures were incubated at 54°C for 18 h. The DNA was purified using the GenElute bacterial genomic DNA kit (Sigma-Aldrich, St. Louis, MO, USA) according to the Gram-positive bacterial protocol with small modifications (15). The VT154-175 strain has been deposited in the BCCM/LMG bacteria collection as LMG 28822. The 16S rDNA sequence identified the strain as belonging to the *T. taiwanensis* species. Total genomic DNA was subjected to library construction according to the Nextera-XT paired-end (PE) protocol (Illumina, San Diego, CA, USA); insert size between 400 bp and 1.5 kbp). Starting from a single library construction, two different sequencing runs were performed with a HiSeq 2000 sequencer (Illumina) in the paired-end sequencing mode. The obtained sequences were trimmed using Erne (version 1.4, http://erne.sourceforge.net/) (16) software developed by Udine University using the following parameters: minimum value used by Mott-like trimming = 20; minimum mean value to accept a trimmed sequence = 20; minimum sequence length after trimming = 40. The removal of the primers was carried out using cutadapt (−O LENGTH = 10, −m LENGTH = 90) (17). A total of 19,837,516 PE reads comprising a total of 6,706,121,550 sequence bases were obtained after trimming and primers removal. The reads were assembled by the Abyss assembler (18) obtaining 128 contigs with an *N*_50_ of 79,854. Only 72 contigs are longer than 500 bp, and some of them have been generated by the assembler with short stretch of gaps. The splitting of those gaps results in 87 contigs. The contigs were processed by the Abyss scaffolder obtaining 109 scaffolds: 60 scaffolds have size over 500 bp. The total genome length was calculated to be 2,925,911 bp with a 68.5% G+C content with an *N*_50_ of 109,912 and an average scaffold size of 48,789 bp.

### Accession number(s).

This whole-genome shotgun project has been deposited at DDBJ/EMBL/GenBank under the accession number JTKY00000000. The version described in this paper is version JTKY01000000.
